# SARS-CoV-2 interaction with Siglec-1 mediates *trans*-infection by dendritic cells

**DOI:** 10.1038/s41423-021-00794-6

**Published:** 2021-11-15

**Authors:** Daniel Perez-Zsolt, Jordana Muñoz-Basagoiti, Jordi Rodon, Marc Elosua-Bayes, Dàlia Raïch-Regué, Cristina Risco, Martin Sachse, Maria Pino, Sanjeev Gumber, Mirko Paiardini, Jakub Chojnacki, Itziar Erkizia, Xabier Muñiz-Trabudua, Ester Ballana, Eva Riveira-Muñoz, Marc Noguera-Julian, Roger Paredes, Benjamin Trinité, Ferran Tarrés-Freixas, Ignacio Blanco, Victor Guallar, Jorge Carrillo, Julià Blanco, Amalio Telenti, Holger Heyn, Joaquim Segalés, Bonaventura Clotet, Javier Martinez-Picado, Júlia Vergara-Alert, Nuria Izquierdo-Useros

**Affiliations:** 1 IrsiCaixa AIDS Research Institute, 08916 Badalona, Spain; 2 IRTA, Centre de Recerca en Sanitat Animal (CReSA, IRTA-UAB), Campus de la UAB, 08193 Bellaterra (Cerdanyola del Vallès), Spain; 3 CNAG-CRG, Centre for Genomic Regulation (CRG, Barcelona Institute of Science and Technology (BIST), 08028 Barcelona, Spain; 4 Centro Nacional de Biotecnología, CSIC, 28049 Madrid, Spain; 5 Division of Microbiology and Immunology, Yerkes National Primate Research Center, Emory University, Atlanta, GA USA; 6 Department of Pathology and Laboratory Medicine, School of Medicine, Emory University, Atlanta, Georgia USA; 7 Division of Pathology, Yerkes National Primate Research Center, Emory University, Atlanta, Georgia USA; 8 Germans Trias i Pujol Research Institute (IGTP), Can Ruti Campus, 08916 Badalona, Spain; 9 Barcelona Supercomputing Center (BSC), 08034 Barcelona, Spain; 10 Catalan Institution for Research and Advanced Studies (ICREA), 08010 Barcelona, Spain; 11 Present Address: University of Vic–Central University of Catalonia (UVic-UCC), Vic, 08500 Spain; 12 Department of Integrative Structural and Computational Biology, Scripps Research, La Jolla, CA 92037 USA; 13 Universitat Pompeu Fabra (UPF), Barcelona, Spain; 14 UAB, CReSA (IRTA-UAB), Campus de la UAB, 08193 Bellaterra (Cerdanyola del Vallès), Spain; 15 Departament de Sanitat i Anatomia Animals, Facultat de Veterinària, UAB, 08193 Bellaterra (Cerdanyola del Vallès), Spain

**Keywords:** Viral infection, Mechanisms of disease

Antigen-presenting cells (APCs) may be resistant to SARS-CoV-2 infection but still contribute to viral pathogenesis. Lectins such as sialic acid-binding Ig-like lectin 1 (Siglec-1/CD169) mediate the attachment of viruses to APCs. Here, we show that APCs effectively capture SARS-CoV-2 within compartments via recognition of Siglec-1. This receptor interacts with sialylated gangliosides on membranes of SARS-CoV-2 variants, as previously shown for retroviruses or filoviruses [1]. Blockage of Siglec-1 on monocyte-derived dendritic cells (MDDCs) decreased SARS-CoV-2 viral transfer or *trans*-infection to bystander target cells. However, monocyte-derived macrophages (MDMs) capturing SARS-CoV-2 via Siglec-1 did not transmit infectious particles. The presence of pulmonary APCs co-expressing Siglec-1 and SARS-CoV-2 corroborated these findings in vivo.

We used the methodology described in the Supplementary Methods to dissect the contribution of Siglec-1 to SARS-CoV-2 pathogenesis. Siglec-1 expression is induced on APCs upon IFN-α or LPS exposure and increased in myeloid cells of COVID-19 patients [2]. Here, we tested whether this lectin could bind SARS-CoV-2 via recognition of sialylated gangliosides on viral membranes. APCs captured incoming SARS-CoV-2 in cellular compartments, eventually leading to viral degradation (Supplementary Fig. 1,2). Raji B cell lines transfected with different lectins (Supplementary Fig. 3A) were pulsed with SARS-CoV-2, washed and assessed by ELISA to measure cell-associated viral nucleocapsid content (Fig. 1A). While Raji cells trasnfected with wild-type Siglec-1 captured SARS-CoV-2, cells transfected with DC-SIGN, Siglec-5, Siglec-7 or devoid of these lectins did not (Fig. 1A). Viral uptake via Siglec-1 relied on the recognition of sialylated ligands, as observed with Raji cells transfected with the Siglec-1 mutant R116A, which lacks sialic acid recognition capacity and did not trap SARS-CoV-2 (Fig. 1A). Raji cells pretreated with the α-Siglec-1 monoclonal antibody (mAb) 7-239 reduced SARS-CoV-2 uptake (Supplementary Fig. 3B). SARS-CoV-2 variants were equally trapped via Siglec-1 but not by the mutated Siglec-1 R116A, indicating that sialic acid recognition is critical (Fig. 1B). Superresolution microscopy of SARS-CoV-2 confirmed that GM1, one of the sialyllactose-containing gangliosides interacting with Siglec-1 [1], was detectable on 74% of virus particles (Fig. 1C). MDMs and MDDCs treated with IFN-α to induce Siglec-1 expression displayed higher SARS-CoV-2 uptake than nonactivated cells (Fig. 1D), and uptake was blocked by the α-Siglec-1 mAb 7-239 (Fig. 1E). These complementary approaches identified Siglec-1 as a central molecule mediating SARS-CoV-2 uptake via sialic acid recognition.

**Fig. 1:**
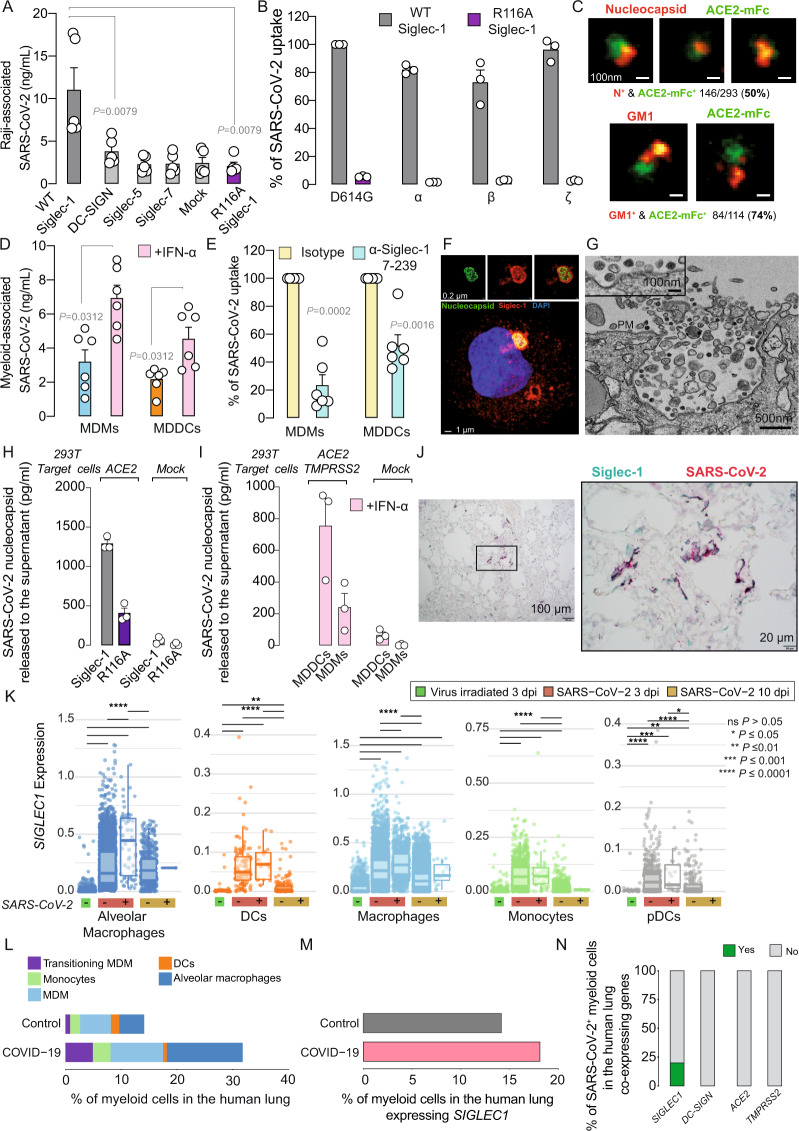
SARS-CoV-2 trapped via Siglec-1 on MDDCs mediates viral *trans*-infection of target cells, and in pulmonary tissues, SARS-CoV-2 is detected on APCs expressing Siglec-1. **A** Uptake of SARS-CoV-2 by Raji B cells measured by ELISA from two experiments. **B** Percentage of SARS-CoV-2 variant uptake from one experiment. Values are normalized to D614G strain uptake by Siglec-1 cells, set at 100%. **C** Superresolution microscopy of SARS-CoV-2 stained with α-nucleocapsid or α-GM1 antibodies (red) and ACE2-mFc (green). The percentage of costaining is shown. **D** Uptake of SARS-CoV-2 by MDMs and MDDCs activated or not with IFN-α for 48 h from two experiments. **E** Percentage of viral uptake inhibition of IFN-α-treated APCs preincubated with mAbs from two experiments. Values are normalized to viral uptake by isotype-treated cells, set at 100%. **F**, **G** Confocal and electron microscopy of VCCs in LPS- and IFN-α-activated MDDCs exposed to SARS-CoV-2. See Movie 1. PM, plasma membrane. **H**, **I** Transmission of SARS-CoV-2 by indicated cells. APCs exposed to SARS-CoV-2 were cocultured with HEK-293T cells expressing ACE2 or not. Viral release from one experiment was measured with an ELISA. **J** Costaining with α-Siglec-1 and α-SARS-CoV-2 nucleocapsid antibodies in the lungs of an infected rhesus macaque. **K** *SIGLEC1* expression on AGM APCs containing or not containing SARS-CoV-2 RNA. Boxplots show the median, the first and third quartiles and whiskers. **L**, **M** Single-cell analysis of pulmonary myeloid cells of 16 COVID-19 patients and 3 controls and APCs expressing *SIGLEC1*. **N** Myeloid cells of COVID-19 patients containing viral RNA and coexpressing *SIGLEC1, DC-SIGN, ACE2* or *TMPRSS2*. **A**, **B**, **D**, **E**, **H,** and **I** show mean values and SEMs. Statistical differences were assessed with the Mann–Whitney *t* test (**A**), Wilcoxon-matched paired *t* test (**D**), one-sample *t* test (**E**), Mann–Whitney–Wilcoxon test with Bonferroni correction (**K**), scCODA (**L**), chi-square test (**M**; *P* = 1.045e^−11^) or Bonferroni-adjusted chi-square test (**N**; *P* = 0.0272624)

Once Siglec-1 binds to HIV-1 or Ebola viruses, receptors polarize and engulf particles within viral-containing compartments (VCCs) that are continuous with the plasma membrane and connected to the extracellular space [1]. To elucidate whether Siglec-1 also recruits SARS-CoV-2 to VCCs, we used confocal and electron microscopy and found Siglec-1-positive VCCs containing viral particles on activated MDDCs (Fig. 1F, G and Movie 1). Siglec-1 has a dual role in enhancing infectivity, either facilitating fusion on APCs, as is the case for the Ebola virus, or mediating transmission to other target cells in *trans*, as is the case for retroviruses [1]. Since SARS-CoV-2 fusion on APCs is limited (Supplementary Fig. 1B), we explored the relevance of viral *trans*-infection. Raji cells were exposed to SARS-CoV-2, washed and cocultured with targets expressing or not expressing ACE2 (Fig. 1H). Raji Siglec-1 cells cocultured with ACE2-expressing cells released higher amounts of SARS-CoV-2 than cells expressing the R116A mutant (Fig. 1H). A minimal viral amount was detected in cocultures with cells lacking ACE2, excluding SARS-CoV-2 replication on Raji cells or the release of initially trapped viruses (Fig. 1H). IFN-α-treated MDDCs cocultured with ACE2- and TMPRSS2-expressing target cells released higher amounts of SARS-CoV-2 than MDMs (Fig. 1I). Experiments with nonreplicative HIV-1 pseudotyped with SARS-CoV-2 spike confirmed Siglec-1 transmission of SARS-CoV-2 via ACE2-dependent fusion on target cells (Supplementary Fig. 4). Hence, SARS-CoV-2 retention on MDDCs via Siglec-1 allows *trans*-infection.

Lung immunohistochemistry analysis from a SARS-CoV-2-infected rhesus macaque used in a previous study [3] confirmed the codetection of Siglec-1 and viral nucleocapsid in cells with myeloid morphology (Fig. 1J). Single-cell RNA sequencing data previously collected in African green monkeys (AGMs) allowed us to focus on pulmonary APCs (Supplementary Fig. 5), where SARS-CoV-2 infection upregulated *SIGLEC1* expression 3 days post-inoculation (dpi) (Supplementary Fig. 6A). Viral RNA was detected in high numbers at 3 dpi, but the levels decreased at 10 dpi (Supplementary Fig. 6B), consistent with the resolution of infection in this mild COVID-19 model. When we compared the expression of *SIGLEC1* on APCs without or with associated viral RNA, only alveolar and interstitial macrophages containing viral RNA significantly increased *SIGLEC1* expression (Fig. 1K). Thus, infection triggers *SIGLEC1* expression on all APCs, but this phenomenon is not linked to the detection of cell-associated viral RNA. We next analyzed the most detailed single-cell atlas [4] of human lungs of COVID-19 patients with advanced disease and controls (Supplementary Fig. 7A-B). Here, APCs were enriched for viral RNA but lacked ACE2 and TMPRSS2 viral entry receptors (Supplementary Fig. 7C). Pulmonary myeloid cells were enriched in COVID-19 patients as compared to those in controls (Fig. 1L; Supplementary Fig. 8A–E), and *SIGLEC1*-expressing APCs were also enriched (Fig. 1M; Supplementary Fig. 8F). Twenty percent of pulmonary APCs expressing viral RNA also coexpressed *SIGLEC1*, whereas none coexpressed other lectins or viral receptors (Fig. 1N). These results corroborated the presence of the Siglec-1 receptor or transcripts on APCs containing SARS-CoV-2 in vivo. Targeting Siglec-1 or *trans*-infection [5] could offer cross-protection against SARS-CoV-2 and other enveloped viruses that exploit APCs for viral dissemination and lead to the development of new broad-spectrum antivirals for future outbreaks.

## Supplementary information

Supplemental Figure Legends

Supplemental Methods

Movie 1

Supplemental Figures
