# Supplementary material for: SARS-CoV-2 interaction with Siglec-1 mediates trans-infection by dendritic cells
Source: Cell Mol Immunol. 2021 Nov 15;18(12):2676–8. doi: 10.1038/s41423-021-00794-6 (PMC8591443; doi:10.1038/s41423-021-00794-6)
Supplement: Supplementary file 1 — Supplemental Figure Legends [file 41423_2021_794_MOESM1_ESM.docx]

***SUPPLEMENTARY FIGURES and MOVIE***

**Supplementary Figure 1.** **Myeloid cells capture and degrade trapped SARS-CoV-2.** **A**. Reduction in the viability of SARS-CoV-2 susceptible Vero E6 cells 3 days post-infection is not observed for monocyte-derived myeloid cells. Percentage of cellular viability 3 days after infection with SARS-CoV-2 at an MOI of 0.1 in Vero E6 cells, MDM and MDDCs. Values from 2 replicates and 4 experiments. Statistical differences from 100% of viability set in the absence of virus were assessed with a one-sample t-test. **B**. Decreased fusion of SARS-CoV-2 spike pseudotyped lentivirus compared to VSV glycoprotein on monocyte-derived myeloid cells. Fusion of HIV-1 luciferase reporter viruses lacking the envelope glycoprotein pseudotyped with VSV glycoprotein or SARS-CoV-2 spike in MDM and MDDCs stimulated or not with IFN-α and in ACE2-expressing HEK-293T cells. ACE2-mFc fusion protein was used to block ACE2-dependent viral fusion. Values from 3 replicates and one experiment. **C**. Fate of trapped viruses on APCs over time. Uptake of SARS-CoV-2 by MDM (left graph) and MDDCs (right graph) activated or not with IFN-α that were pulsed for 4 h, 24 h and 48 h at 37 °C with the virus, to assess the amount of virus present in the supernatant (squares), and the amount of cell-associated viral nucleocapsid detected on cellular lysates after extensive washing (circles) by ELISA. Data from one representative experiment out of two shows means and SEM from 3 different donors. **D**. Kinetics of SARS-CoV-2 degradation after 4 h of viral exposure and extensive washing by MDM (left graph) and MDDCs (right graph) activated or not with IFN-α. The amount of virus present in the supernatant (squares) and the amount of cell-associated viral nucleocapsid detected on cellular lysates after extensive washing (circles) was measured by ELISA. Data from one representative experiment out of two shows means and SEM from 3 different donors.

**Supplementary Figure 2. Fate of trapped SARS-CoV-2 on activated APCs analyzed by electron microscopy.** Cells were exposed to SARS-CoV-2 at an MOI of 1. **A**. Extracellular viruses attached to the plasma membrane. **B** and **E**. Extracellular viruses in vacuoles connected to the plasma membrane outside the plane of the section **C**. Viruses in early sorting endosomes. **D** and **F**. Large membranous compartments resembling degradative structures such as lysosomes, where damaged viral particles were observed. **G** and **K**. Extracellular virus attached to the plasma membrane. **H.** Membranous VCC continuous with the plasma membrane. **I-J** Lysosomes with different viral particles and membranes in the lumen undergoing degradation **L-M**. Vesicles filled with viral particles and material that marks them as endocytic structures. Arrows indicate individual viral particles. PM, plasma membrane; EE, Early endosome; L, Lysosome; M, mitochondrion. Data from 2 donors and 2 experiments.

**Supplementary Figure 3. SARS-CoV-2 uptake by Raji B cell lines transfected with different lectins. A.** Representative surface staining of the different lectins expressed on transfected Raji cell lines analyzed by FACS. **B**. Uptake of SARS-CoV-2 by Raji Siglec-1 pre-incubated with α-Siglec-1 mAb 7-239 or the corresponding isotype control, that were pulsed for 2 h at 37°C, washed and lysed to assess the amount of cell-associated viral nucleocapsid by ELISA. Values from three replicates and one experiment.

**Supplementary Figure 4. SARS-CoV-2 trapped via Siglec-1 on DCs mediates viral *trans*-infection of target cells. A**. Transmission of SARS-CoV-2 pseudoviruses from distinct Raji cells to the indicated target HEK-293T cells expressing ACE2 or ACE2 and TMPRSS2. Raji cells were pulsed with an HIV-1 luciferase reporter virus lacking the envelope glycoprotein pseudotyped with SARS-CoV-2 Spike for 2 h. Cells were washed and co-cultured with target cells for 48 h. Infection of target cells was determined by induced luciferase activity in relative light units (RLUs). No viral fusion was detected on Raji cells cultured alone. Data show mean values and SEMs from two experiments including three replicates. Statistical differences were assessed with a Mann Whitney test. **B**. Transmission of SARS-CoV-2 pseudoviruses captured for 4 h from MDDCs and MDM to HEK-293T cells expressing ACE2 and TMPRSS2. Data show mean values and SEMs from two experiments including cells from six donors. Statistical differences were assessed with a paired t test. **C**. Transmission of SARS-CoV-2 pseudoviruses from IFN-α activated MDDCs to HEK 293T cells expressing ACE2 and TMPRSS2. Cells were pre-incubated with the indicated mAbs and exposed to SARS-CoV-2 before assessing *trans*-infection. No viral fusion was detected on MDDCs when cultured in absence of SARS-CoV-2 target cells. Data show mean values and SEMs from two experiments including cells from six donors. Statistical differences were assessed with a paired t test.

**Supplementary Figure 5. Single cell RNA sequencing analysis of AGM. A.** Uniform Manifold Approximation and Projection embedding of the pulmonary cells from SARS-CoV-2-inoculated AGM analyzed by scRNAseq colored by their annotated cell type. **B**. DotPlot showing the marker gene expression for each cell type.

**Supplementary Figure 6**. **SARS-CoV-2 is detected on APCs expressing Siglec-1 in pulmonary tissues.** **A**. *SIGLEC-1* expression detected using single cell RNA sequencing (scRNAseq) on APCs of AGMs sacrificed after 3 dpi with irradiated virus, or sacrificed after 3 or 10 dpi with SARS-CoV-2. Data from 12 animals. **B**. SARS-CoV-2 expression detected as in **A**, shown as a gene signature encompassing all viral RNA. Data from 12 animals.

**Supplementary Figure 7**. **Single cell RNA sequencing analysis of pulmonary cells in humans.** **A**. Uniform Manifold Approximation and Projection embedding of the pulmonary cells from SARS-CoV-2 infected individuals analyzed by scRNAseq colored by their annotated cell type. **B**. DotPlot showing the marker gene expression for each cell type. **C**. Uniform Manifold Approximation and Projection embedding of the human pulmonary cells analyzed by scRNAseq colored by the expression of the transcripts *ACE2*, *TMPRSS2*, *SIGLEC1* and SARS-CoV-2 RNA.

**Supplementary Figure 8**. **APCs express Siglec-1 in pulmonary human cells. A**. Uniform Manifold Approximation and Projection embedding of the myeloid pulmonary cells from SARS-CoV-2 infected individuals analyzed by scRNAseq colored by their annotated cell type. **B**. DotPlot showing the marker gene expression for each cell type. **C.** Uniform Manifold Approximation and Projection embedding of the human pulmonary myeloid cells analyzed by scRNAseq, colored by their annotated cell type in control *versus* SARS-CoV-2-infected individuals. **D**. scCODA differential abundance analysis of the myeloid cell subtypes in control *versus* SARS-CoV-2-infected individuals. **E**. Percentage of myeloid cells in the lungs of controls and SARS-CoV-2 infected individuals shown per patient. **F.** Uniform Manifold Approximation and Projection embedding of the human myeloid pulmonary cells analyzed by scRNAseq and colored by the expression of the *SIGLEC1* transcript in control *versus* SARS-CoV-2-infected individuals*.*

**Movie 1**. **SARS-CoV-2 is detected on VCCs of MDDCs containing Siglec-1.** Deconvolved 3D reconstruction of an LPS-treated MDDC exposed to SARS-CoV-2 showing a VCC. Green; anti-nucleocapsid pAbs, Red; anti-Siglec-1 mAb, and Blue; DAPI staining the nucleus.
