# Supplementary material for: SARS-CoV-2 interaction with Siglec-1 mediates trans-infection by dendritic cells
Source: Cell Mol Immunol. 2021 Nov 15;18(12):2676–8. doi: 10.1038/s41423-021-00794-6 (PMC8591443; doi:10.1038/s41423-021-00794-6)
Supplement: Supplementary file 2 — Supplemental Methods [file 41423_2021_794_MOESM2_ESM.docx]

***MATERIAL AND METHODS***

***Ethics statement.*** The institutional review board on biomedical research from Hospital Germans Trias i Pujol (HUGTiP) approved this study. The biologic biosafety committee of the Research Institute Germans Trias i Pujol approved the execution of SARS-CoV-2 experiments at the BSL3 laboratory of the Centre for Comparative Medicine and Bioimage (CMCiB).

***Cell cultures****.* Vero E6 cells (ATCC CRL-1586) were cultured in Dulbecco’s modified Eagle medium, (DMEM; Lonza) supplemented with 5% fetal bovine serum (Invitrogen), 100 U/mL penicillin, 100 µg/mL streptomycin, and 2 mM glutamine (all ThermoFisher Scientific). HEK-293T (ATCC repository) were maintained in DMEM with 10% fetal bovine serum, 100 IU/mL penicillin and 100 μg/mL streptomycin (all from Invitrogen). Raji B lymphocyte and Raji DC-SIGN cell lines (kindly provided by Y. Van Kooyke) were maintained in Roswell Park Memorial Institute medium (RPMI; Invitrogen) or RPMI plus 1 mg/mL geneticin (Invitrogen). Generation and maintenance of Raji Siglec-1, Raji R116A, Raji Siglec-5 and Raji Siglec-7 has been described elsewhere ^1^. HEK-293T overexpressing the human ACE2 were kindly provided by Integral Molecular Company and maintained in DMEM with 1 μg/mL of puromycin (Invitrogen). TMPRSS2 human plasmid (Origene) was transfected using X-tremeGENE HP Transfection Reagent (Merck) on HEK-293T overexpressing the human ACE2 and maintained in the previously described media containing 1 mg/mL of geneticin (Invitrogen) to obtain TMPRSS2/ACE2 HEK-293T cells. All media contained 10% fetal bovine serum, 100 IU/mL penicillin and 100 μg/mL streptomycin (all from Invitrogen).

***Primary cell cultures****.* Peripheral blood mononuclear cells were obtained with a Ficoll-Hypaque gradient (Alere Technologies AS) from blood donors and monocyte populations (>90% CD14^+^) were isolated with CD14-negative selection magnetic beads (Miltenyi Biotec). Monocyte derived macrophages (MDM) were obtained culturing these cells in the presence of 100 µg/mL of macrophage colony-stimulating factor (M-CSF) for seven days and replacing media and cytokines every 2 days. Monocyte derived DCs (MDDCs) were obtained culturing these cells in the presence of both 1,000 IU/mL of granulocyte-macrophage colony-stimulating factor (GM-CSF) and interleukin-4 (IL-4; both from R&D) for seven days and replacing media and cytokines every 2 days. Activated cells were differentiated by culturing myeloid cells at day five for two more days in the presence of 1,000 IU/mL of interferon-alfa (IFN-α; Sigma-Aldrich) or 100 ng/mL of lipopolysaccharide (LPS, Sigma-Aldrich).

***Virus isolation, titration and sequencing****.* Unless otherwise specified, SARS-CoV-2 used was the virus isolated in March 2020 from a nasopharyngeal swab as described in ^2^. The virus was propagated for two passages and a virus stock was prepared collecting the supernatant from Vero E6. Genomic sequence was deposited at GISAID repository (<http://gisaid.org>) with accession ID EPI_ISL_510689. Compared to the Wuhan/Hu-1/2019 strain, this isolate has the following point mutations: 376 D614G (spike), R682L (spike), and C16X (NSP13). The SARS-CoV-2 Alpha or B.1.1.7 variant (originally isolated from the UK), the Gamma or B.1.1.248.2 variant (originally isolated from Brazil) and the Beta pr B.1.351 variant (originally isolated from South Africa) were identified during routine sequencing of a clinical nasopharyngeal swabs in Spain during January-February 2021 and subsequently isolated on Vero E6 cells. These sequences are deposited at GISAID database with accession numbers EPI_ISL_1663569; EPI_ISL_1831696 and EPI_ISL_1663571 for B.1.17, B.1.1.248.2 and B.1.351, respectively. Genomic sequencing was performed from viral supernatant by using standard ARTIC v3 based protocol followed by Illumina sequencing (dx.doi.org/10.17504/protocols.io.bhjgj4jw). Raw data analysis was performed by viralrecon pipeline (<https://github.com/nf-core/viralrecon>) while consensus sequence was called using samtools/ivar at the 75% frequency threshold.

***Pseudovirus production****.* HIV-1 reporter pseudoviruses expressing SARS-CoV-2 spike protein and luciferase were generated using two plasmids. pNL4-3.Luc.R-.E- was obtained from the NIH AIDS repository. SARS-CoV-2.SctΔ19 was generated (Geneart) from the full protein sequence of SARS-CoV-2 spike with a deletion of the last 19 amino acids in the C-terminal, human-codon optimized and inserted into pcDNA3.4-TOPO ^3^. Spike plasmid was transfected with X-tremeGENE HP Transfection Reagent (Merck) into HEK-293T cells, and 24 h post-transfection, cells were transfected with pNL4-3.Luc.R-.E-. VSV-g plasmid (kindly provided by A. Cimarelli) was used to equally pseudotype pseudoviruses. Supernatants were harvested 48 h later, filtered with 0.45 µM (Millex Millipore) and stored at -80ºC until use. Viruses were titrated in HEK-293T overexpressing the human ACE2.

***Pseudoviral fusion assay.*** MDM or MDDCs activated or not with IFN-α as previously described along with HEK-293T ACE2 cells were exposed to equivalent MOI of VSVg or SARS-CoV-2 spike pseudotyped lentiviruses. To block ACE2 dependent viral fusion, some wells had 20 µg/mL of human ACE2-murine Fc fusion protein. Two days post-infection, cells were lysed with the Glo Luciferase system (Promega). Luminescence was measured with an EnSight Multimode Plate Reader (Perkin Elmer).

***Construction of a human-ACE2 murine-Fc-fusion protein (ACE2-mFc****)*. Expression vector was generated with the Geneart service (ThermoFisher Scientific). Coding sequence included the first 615 amino acids from the Human ACE2 sequence, with H345A and H505A mutations to inactivate the catalytical sites, followed by the constant region of the heavy chain of the murine IgG1. For protein production, Expi293F cells (ThermoFisher Scientific) were transfected with ACE2-mFc vector at a density of 2.5 x 10^6^ cells/mL using Expifectamine (ThermoFisher Scientific). Enhancers 1 and 2 (ThermoFisher Scientific) were added to the culture 18 h post-transfection. Cells were incubated for 5 days and supernatants were harvested and passed through a 0.22 µm PVDF filter. For purification, supernatants were loaded into a 5 mL SepFast Ø11mm (Quimigen) packed with CaptureSelect™ IgG-Fc (ms) affinity resin (ThermoFisher Scientific) connected to an Äkta Start Chromatograph (Cytiva). The column was washed with 5 column volumes (CV) of PBS and ACE2-mFc was eluted with 2 CV of 0.1M Glycine at pH=3.5. The sample was concentrated with a 30kDa Amicon Centrifugal Concentrator at 3000 x g. ACE2-mFc concentration was determined by sandwich ELISA using a goat anti-mouse IgG Fc (Jackson Immunoresearch, 115-006-071) for capture, a horseradish peroxidase (HRP) labeled F(ab)2 Goat anti-mouse IgG Fc (Jackson Immunoresearch, 115-036-071) as secondary antibody, A purified mouse IgG (D50, NIH AIDS Reagent Program) as standard and o-phenylenediamine dihydrochloride (Sigma-Aldrich, #P8787-100TAB) as substrate. Light absorbance was measured at 492/620 nm on EnSight Multimode Plate Reader (Perkin Elmer). ACE2-mFc inhibitory capacity was tested in a SARS-CoV-2 neutralization assay as described previously ^4^.

***SARS-CoV-2 uptake and degradation assays****.* Uptake experiments with SARS-CoV-2 were performed pulsing 0.5x10^6^ Raji or 1x10^6^ myeloid cells at a rate of 70 ng of nucleocapsid at 37ºC for the indicated timepoints. For blockade, cells were pre-incubated for 15 min at room temperature (RT) with 10 μg/mL of α-Siglec-1 7–239 monoclonal antibody (mAb), or IgG1 isotype control (BD Biosciences), or left untreated before viral exposure. After extensive washing, cells were lysed at a constant concentration of 1x10^6^ cells/mL, centrifuged to remove cellular debris and assayed with a SARS-CoV-2 nucleocapsid protein (NP) High-sensitivity Quantitative ELISA (ImmunoDiagnostics). For degradation experiments, myeloid cells were exposed to SARS-CoV-2 for 4 h, extensively washed, and left in culture for the indicated timepoints until cell associated viral content and viral release to the supernatant were measured with the indicated ELISA kit.

***Electron microscopy of myeloid cells***. 10x10^6^ myeloid cells (MDM or MDDCs activated with LPS or IFN-α) were exposed to SARS-CoV-2 with an MOI of 1 for 24 h, fixed with paraformaldehyde (PFA) at 4% (Biotium) and glutaraldehyde 1% (Sigma Aldrich/Merck) for 1 h at RT and processed for embedding in resin, ultramicrotomy and transmission electron microscopy as previously described ^5^. Briefly, after fixation the samples were washed three times with PBS and cells were gently scraped with a rubber policeman. Cell pellets were postfixed with 1% osmium tetroxide + 0.8% potassium ferrocyanide in water for 1 h on ice. The samples were dehydrated on ice with a gradual series of acetone and infiltrated at RT with epoxy resin. After heat polymerization the samples were sectioned with a UC6 microtome with a nominal feed of 70 nm. Sections were collected on 300 mesh bare copper grids and contrasted with 4% aqueous uranyl acetate, followed by Reynold´s lead citrate. Images were taken either using a Jeol 1011 run at 100 kV equipped with a Gatan ES1000W camera or a Jeol 1400 run at 80kV with a Gatan Oneview camera.

***Confocal microscopy analyses***. LPS-treated MDDCs were pulsed with SARS-CoV-2 with an MOI of 1 for 4 h at 37 °C. After extensive washing, cells were fixed and permeabilized (Fix & Perm, Invitrogen) and stained with anti-rabbit nucleocapsid polyclonal antibody (pAb) (GeneTex) revealed with a Goat pAb Anti-Rabbit IgG Alexa 488 (Abcam) and an anti-Siglec-1 7-239 Alexa 647 mAb (Biolegend). Cells were cytospun into coverslips, covered with DAPI-containing Fluoroshield mounting medium (Sigma-Aldrich) and analyzed with a Dragonfly (Andor) 505 multimodal confocal microscope with GPU driven deconvolution to maximize resolution.

***Super-resolution analysis of SARS-CoV-2****.* For super-resolution detection of nucleocapsid and spike proteins and GM1 gangliosides, SARS-CoV-2 particles were adhered to poly-L coated coverslips for 15 min at RT and fixed in 4 % PFA/PBS for 30 min. Fixed and inactivated virus samples were permeabilized and blocked using 0.1 % saponin /0.5 % BSA/PBS. Virus particles were immunostained with rabbit anti-SARS-CoV-2 N protein (Sino Biological) or rabbit anti-GM1 Ab (Abcam) followed by anti-rabbit Abberior STAR RED (Abberior GmbH) Fab fragments. SARS-CoV-2 spike protein was detected with an ACE2- mAb Fc recombinant protein and anti-mouse Abberior STAR 580 (Abberior GmbH) Fab fragments. SARS-CoV-2 spike protein was detected with an ACE2- mAb Fc recombinant protein and anti-mouse Abberior STAR RED conjugated Fab fragments. Following immunostaining, all samples were overlaid with SlowFade Diamond mounting medium (ThermoFisher Scientific) and imaged using STED microscopy. Super-resolution analysis of SARS-CoV-2 virus particles was performed using Leica SP8 STED 3X microscope (Mannheim, Germany) equipped with a 100×/1.4 NA oil immersion STED objective. STED images of N protein, GM1 (Abberior STAR RED) and S-ACE2 protein complexes (Abberior STAR 580) signals were acquired sequentially for each channel using 637 nm and 587 nm lines from the white light laser. Abberior STAR RED and Abberior STAR 580 signal was depleted with a donut-shaped 775-nm pulsed STED laser. STED depletion conditions were tuned to achieve 40 nm lateral resolution (full-width-at-half-maximum, FWHM) as estimated from fluorescent bead and single fluorescent antibody molecule measurements. STED images were acquired with following parameters: pinhole size: 1.03 Airy; dwell time: 2 μs/pixel and XY pixel size: 20 nm. Acquired STED images were thresholded and filtered using Gaussian filter (Sigma (Radius) = 0.75) using Fiji (ImageJ distribution) software.

***Trans-infection assay****.* HEK-293T overexpressing the human ACE2 or lacking this molecule were used to test if SARS-CoV-2 replication competent virus was *trans*-infected. Uptake experiments with SARS-CoV-2 were performed pulsing 0.1x10^6^ Raji cells or IFN-α-activated MDDCs and MDM with an MOI of 2 for 3 h at 37ºC. After extensive washing, cells were co-cultured at a ratio 3:1 with HEK-293T cells expressing or not ACE2. Six days later, supernatant was assayed with a SARS-CoV-2 nucleocapsid protein (NP) High-sensitivity Quantitative ELISA (ImmunoDiagnostics).

HEK-293T overexpressing the human ACE2, or both ACE2 and TMPRSS2 were used to test if SARS-CoV-2 pseudotyped viruses can fuse with these receptors via *trans*-infection. A constant pseudoviral titer was used to pulse Raji cells or IFN-α-activated MDDCs and MDM in the presence of the indicated mAbs at 10 μg/ml for 2 h at 37ºC. Cells were extensively washed and co-cultured with or without target cells at a ratio 1:1. Two days post-infection, cells were lysed with the Bright-Glo luciferase assay system (Promega). Luminescence was measured with an EnSight Multimode Plate Reader (Perkin Elmer).

***Immunohistochemical staining on sections of SARS-CoV-2.*** The paraffin-embedded sections of SARS-CoV-2 infected lungs from a previous study with *Rhesus macaques* ^6^ were subjected to deparaffinization in xylene, rehydration in graded series of ethanol, and rinsed with double distilled water. Antigen retrieval was performed by immersing sections in DIVA Decloaker (Biocare Medical) at 125˚C for 30 sec in a steam pressure decloaking chamber (Biocare Medical) followed by blocking with SNIPER Reagent (Biocare Medical) for 10 min. The sections were incubated with SARS nucleocapsid protein antibody (Rabbit polyclonal; Novus Biologicals, NB100-56576SS) and rabbit anti-human Anti-Sialoadhesin/CD169 antibody (clone SP216, Abcam, ab183356) for 1 h, followed by a double detection polymer system (Mach 2 Double Stain 2, Biocare Medical). Labeled antibodies were visualized by development of the chromogen (Warp Red and/or Vina Green Chromogen Kits; Biocare Medical). Digital images of lung were captured at 100×, 200× and 400× magnification with an Olympus BX43 microscope equipped with a digital camera (DP27, Olympus) and evaluated using Cellsens Standard digital imaging software 2.3 (Olympus).

***Data origin, quality control and data processing of single-cell RNA sequencing methods.*** We used publicly available lung single-cell RNA sequencing data from African green monkey (AGM) *Chlorocebus aethiops* ^7^ in which monkeys were inoculated with infectious SARS-CoV-2 or irradiated, inactivated virus and sacrificed at 3 dpi and 10 dpi. The data originally used by Speranza *et al.* is publicly available in the Gene Expression Omnibus through GEO Series accession no. GSE156755 ([www.ncbi.nlm.nih.gov/geo/query/acc.cgi?acc=GSE156755](http://www.ncbi.nlm.nih.gov/geo/query/acc.cgi?acc=GSE156755)). Data generation protocols and read mapping can be found in the original publication.

Raw data was loaded and processed with R 4.0.1 (R Core Team (2021). R: A language and environment for statistical computing. R Foundation for Statistical Computing, Vienna, Austria. URL <https://www.R-project.org/>) using Seurat V4 ^8^. Processing and quality control steps were carried out as detailed in the original manuscript, all datasets were integrated using Seurat’s IntegrateData function and we then filtered out low quality cells and doublets by removing cells with abnormally mitochondrial genes (greater than 3 SDs above the median), and cells that were likely doublets were relabeled [ratio of unique features to unique mapped identifier (UMI) per cell < 0.15]. Also, cells containing less than or greater than 3 SDs of UMI compared to the population total were removed to filter for noise ^7^. Principal component analysis was carried out in the integrated space and clustering and UMAP embedding was performed using the top 30 principal components. Data normalization was carried out in the raw count matrix space using Seurat’s function SCTransform.

We also used publicly available data from the human lung ^9^ in which they carried out single-cell and single-nuclei RNAseq (sc/snRNAseq) from an autopsy cohort of 20 male and 12 female donors. Lung samples were processed for 16 COVID-19 donors and 3 non-COVID-19 donors. No data preprocessing was carried out since we used the readily available processed data ( publicly available in the Gene Expression Omnibus through GEO Series accession no. GSE171668). The processed data used in this study is also available on the Single Cell Portal: Lung, <https://singlecell.broadinstitute.org/single_cell/study/SCP1052/> .

***Single-cell RNA sequencing downstream analysis and code availability.***

For AGM datasets, cells were annotated using canonical marker genes obtained from the original paper and previously published human lung atlases ^10^. A SARS-CoV-2 gene signature looking at all the viral RNAs was obtained for all the cells and those with a positive score, identifying those with at least one viral RNA, were labeled as SARS-CoV-2-positive. Due to the sparsity of the scRNAseq data we used MAGIC ^11^ denoising on the normalized count matrix to recover the underlying structure of the data and enhance the signal of lowly expressed genes. To statistically test the levels of *SIGLEC1* at the different necropsy timepoints and between SARS-CoV-2^+^ and SARS-CoV-2^-^ we carried out pairwise Mann–Whitney–Wilcoxon tests across all conditions and adjusted the *p*-values using the Bonferroni correction.

With the human sc/snRNAseq data we used the provided UMAP embedding as well as the cell identity annotations. We used scCODA ^12^ to determine enrichment of specific cell types. Chi-square test was carried out to determine the statistical significance between the proportions of myeloid cells in the human lung expressing *SIGLEC1* between controls and COVID-19 patients. We also used the Chi-square test with Bonferroni correction to test the significance of the comparison between the proportions of SARS-CoV-2^+^ cells expressing *SIGLEC1* and those expressing *DC-SIGN*, *ACE2* and *TMPRSS2*. Lastly, we used the Mann–Whitney–Wilcoxon test with Bonferroni correction to compare the expression of key cytokines in COVID-19 donors between *SIGLEC1^+^* and *SIGLEC1^-^* cells by cell identity. All analyses were carried out using R4.0.1 and Python 3.8, data was analyzed using Seurat V4 ^8^. All the code used for the analysis can be found in the GitHub repository <https://github.com/MarcElosua/SIGLEC1-SARS-CoV-2>.

***Statistical analyses of non-single cell data.*** Statistical differences from 100% of viability were assessed with a one-sample *t*-test. Statistical differences were also assessed with a Mann Whitney *t* test, a Wilcoxon matched paired *t* test and a paired *t* test. Comparisons were performed with Graph Prism 9.

***BIBLIOGRAPHY***

1. Perez-Zsolt, D. *et al.* Anti-Siglec-1 antibodies block Ebola viral uptake and decrease cytoplasmic viral entry. *Nat Microbiol* **4**, 1558–1570 (2019).

2. Rodon, J. *et al.* Identification of Plitidepsin as Potent Inhibitor of SARS-CoV-2-Induced Cytopathic Effect After a Drug Repurposing Screen. *Front Pharmacol* **12,** 646676 (2021).

3. Ou, X. *et al.* Characterization of spike glycoprotein of SARS-CoV-2 on virus entry and its immune cross-reactivity with SARS-CoV. *Nat Commun* **11**, 1620 (2020).

4. Trinité, B. *et al.* SARS-CoV-2 infection elicits a rapid neutralizing antibody response that correlates with disease severity. *Sci Rep* **11**, 2608 (2021).

5. Tenorio, R. *et al.* Reovirus σNS and µNS Proteins Remodel the Endoplasmic Reticulum to Build Replication Neo-Organelles. *mBio* **9**, 15 (2018).

6. Hoang, T. N. *et al.* Baricitinib treatment resolves lower-airway macrophage inflammation and neutrophil recruitment in SARS-CoV-2-infected rhesus macaques. *Cell* **184**, 460-475.e21 (2021).

7. Speranza, E. *et al.* Single-cell RNA sequencing reveals SARS-CoV-2 infection dynamics in lungs of African green monkeys. *Sci. Transl. Med.* **13**, eabe8146 (2021).

8. Hao, Y. *et al.* Integrated analysis of multimodal single-cell data. *bioRxiv*. http://biorxiv.org/lookup/doi/10.1101/2020.10.12.335331 (2020) doi:10.1101/2020.10.12.335331.

9. Delorey, T. M. *et al.* COVID-19 tissue atlases reveal SARS-CoV-2 pathology and cellular targets. *Nature* **595**, 107–113 (2021).

10. Travaglini, K. J. *et al.* A molecular cell atlas of the human lung from single-cell RNA sequencing. *Nature* **587**, 619–625 (2020).

11. van Dijk, D. *et al.* Recovering Gene Interactions from Single-Cell Data Using Data Diffusion. *Cell* **174**, 716-729.e27 (2018).

12. Büttner, M., Ostner, J., Müller, CL., Theis, FJ. & Schubert, B. scCODA: A Bayesian model for compositional single-cell data analysis. *bioRxiv*.12.14.422688 (2020) doi:10.1101/2020.12.14.422688.
