# Supplementary material for: SARS-CoV-2 interaction with Siglec-1 mediates trans-infection by dendritic cells
Source: Cell Mol Immunol. 2021 Nov 15;18(12):2676–8. doi: 10.1038/s41423-021-00794-6 (PMC8591443; doi:10.1038/s41423-021-00794-6)
Supplement: Supplementary file 4 — Supplemental Figures [file 41423_2021_794_MOESM4_ESM.pdf]

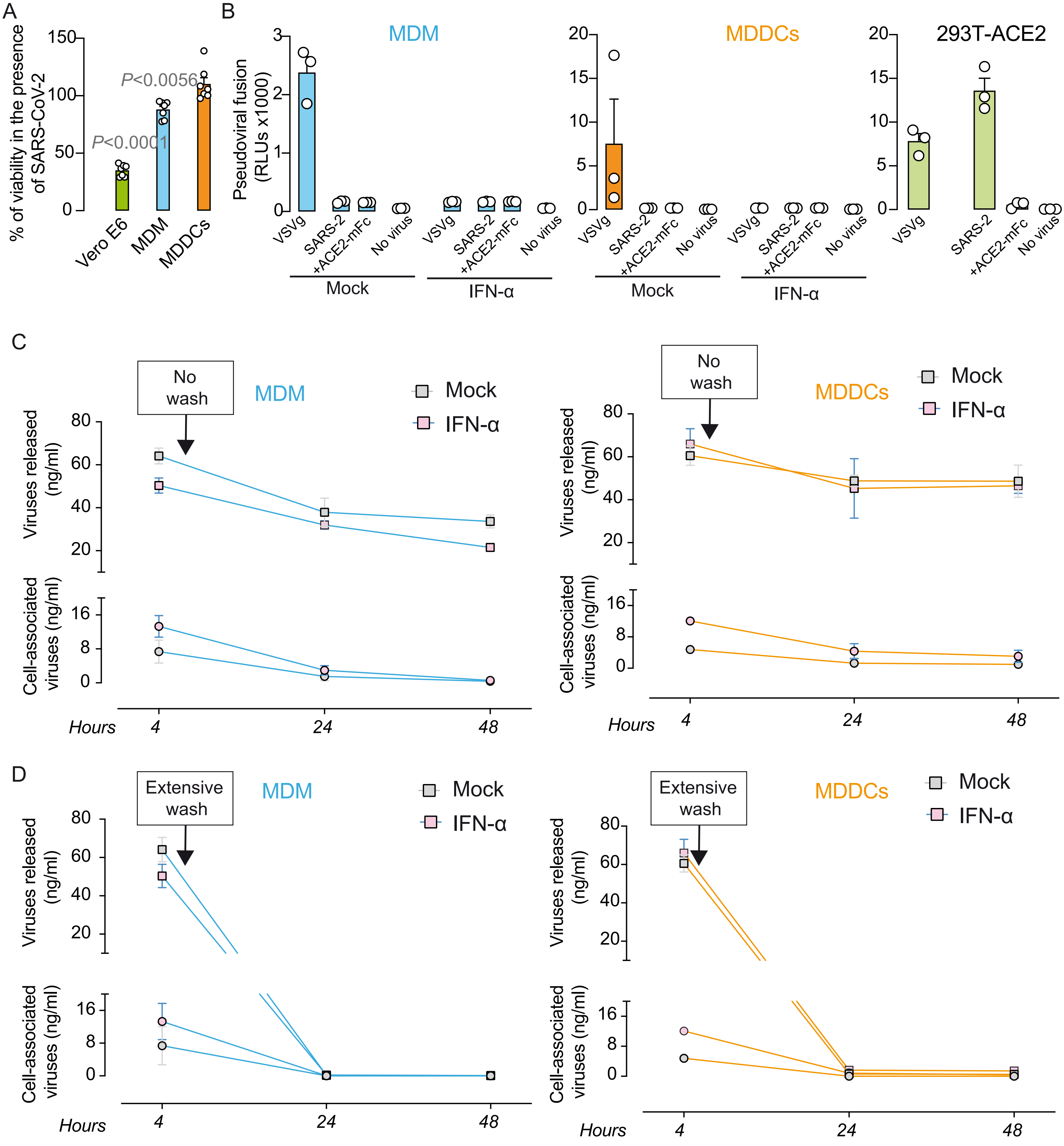

Supplementary Figure 1

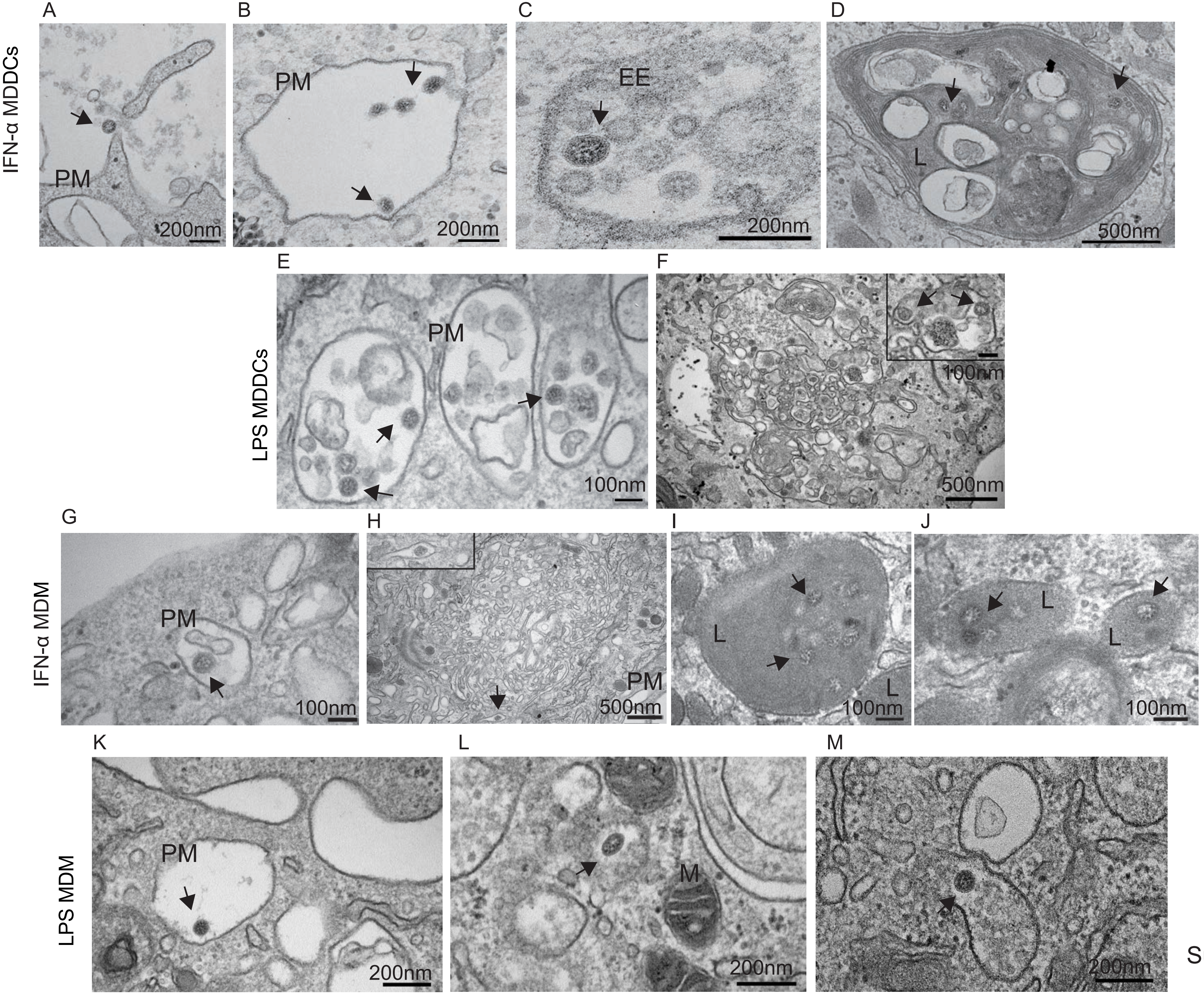

Supplementary Figure 2

A

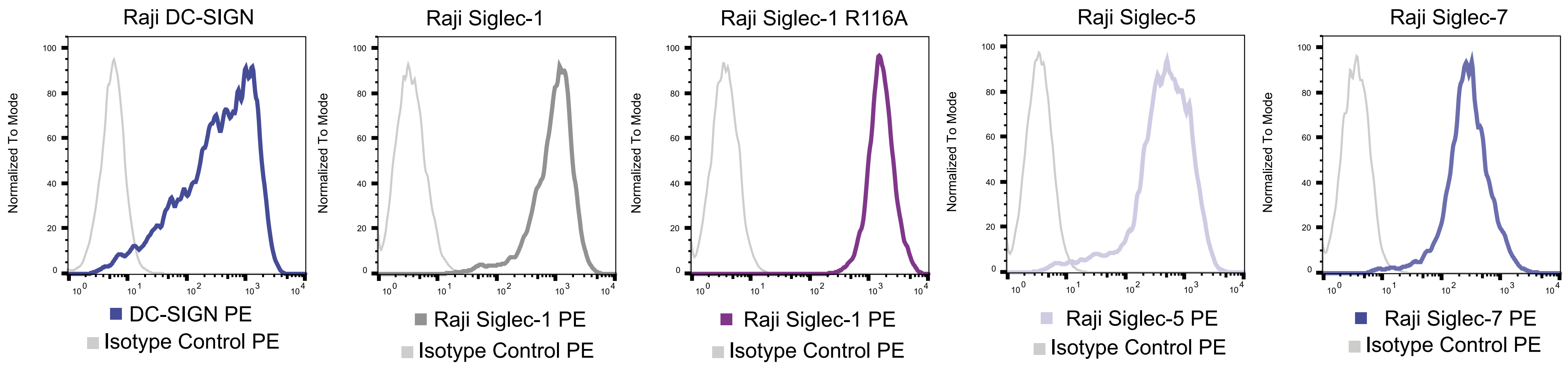

B

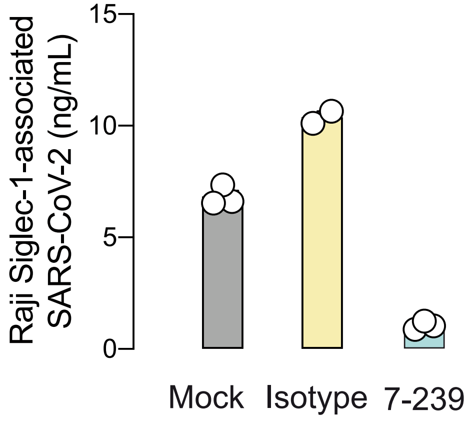

Supplementary Figure 3

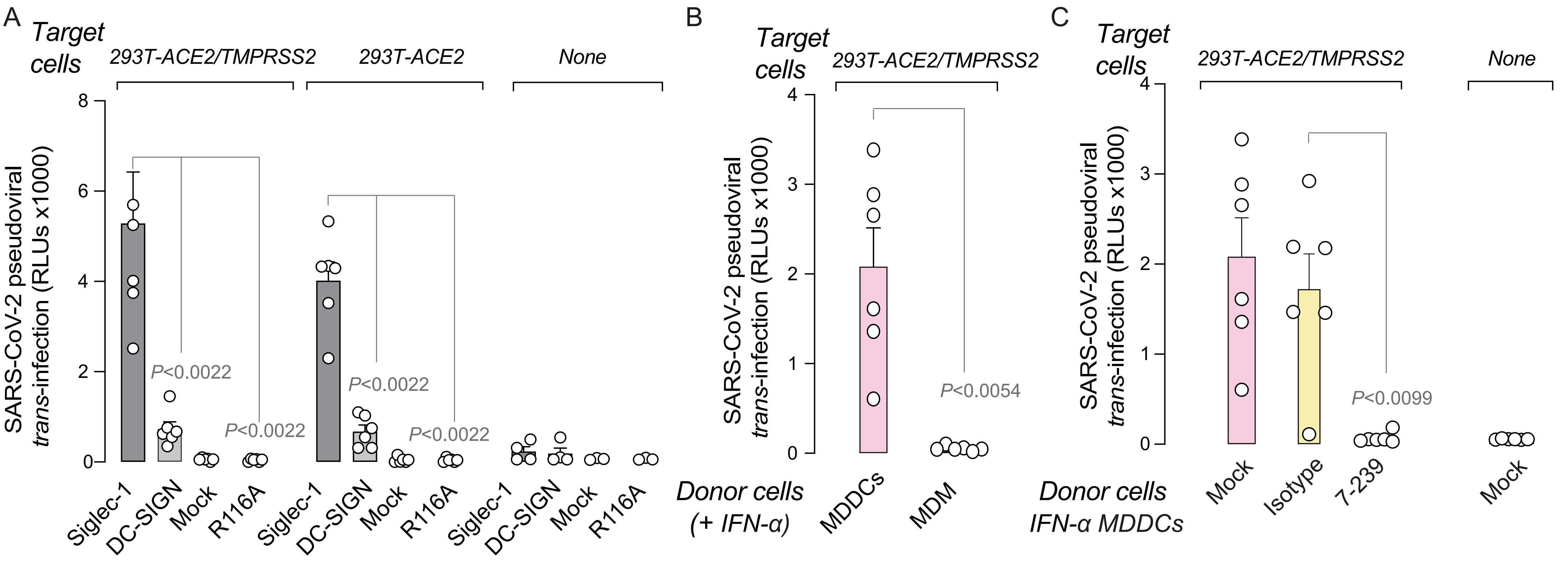

Supplementary Figure 4

A

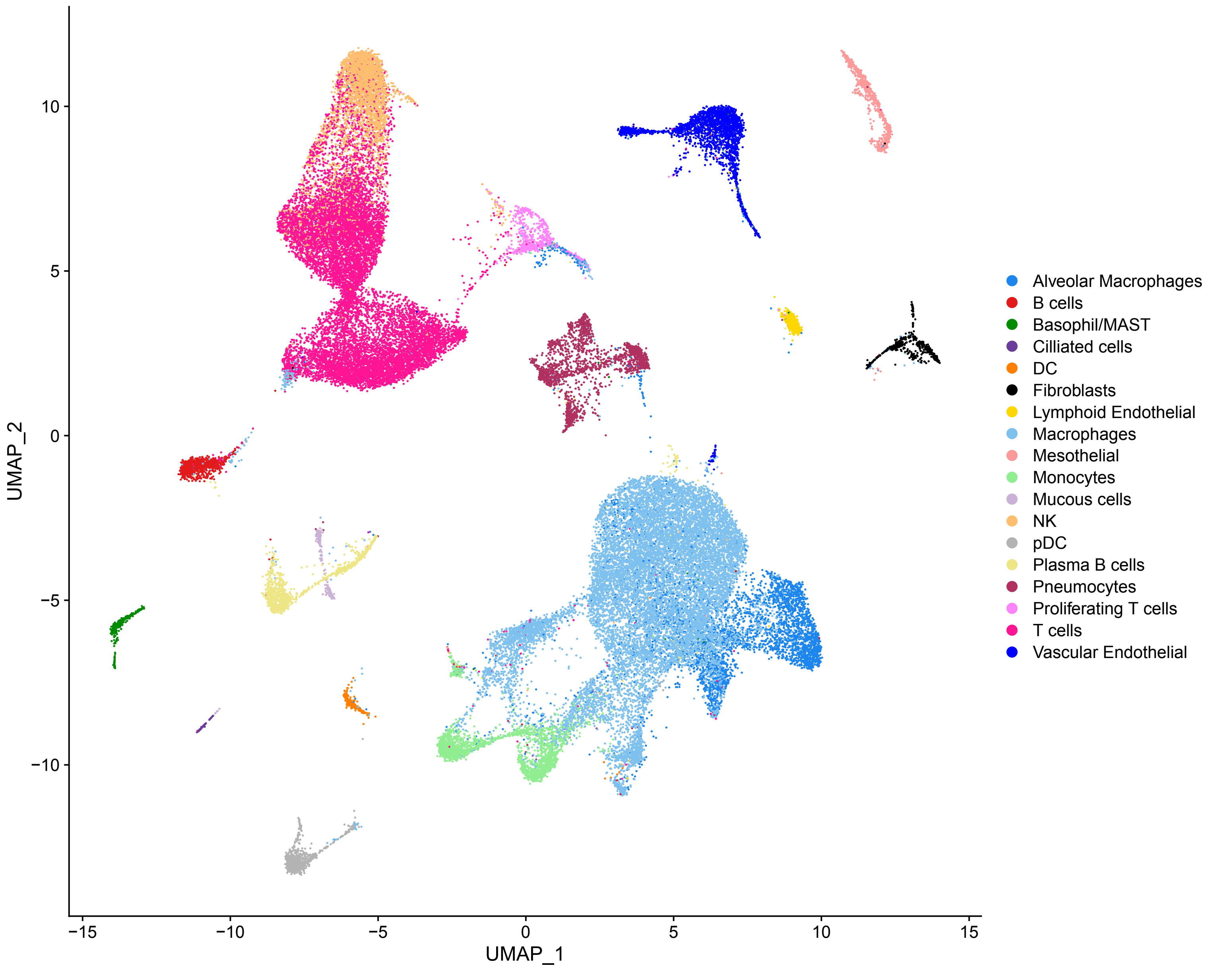

B

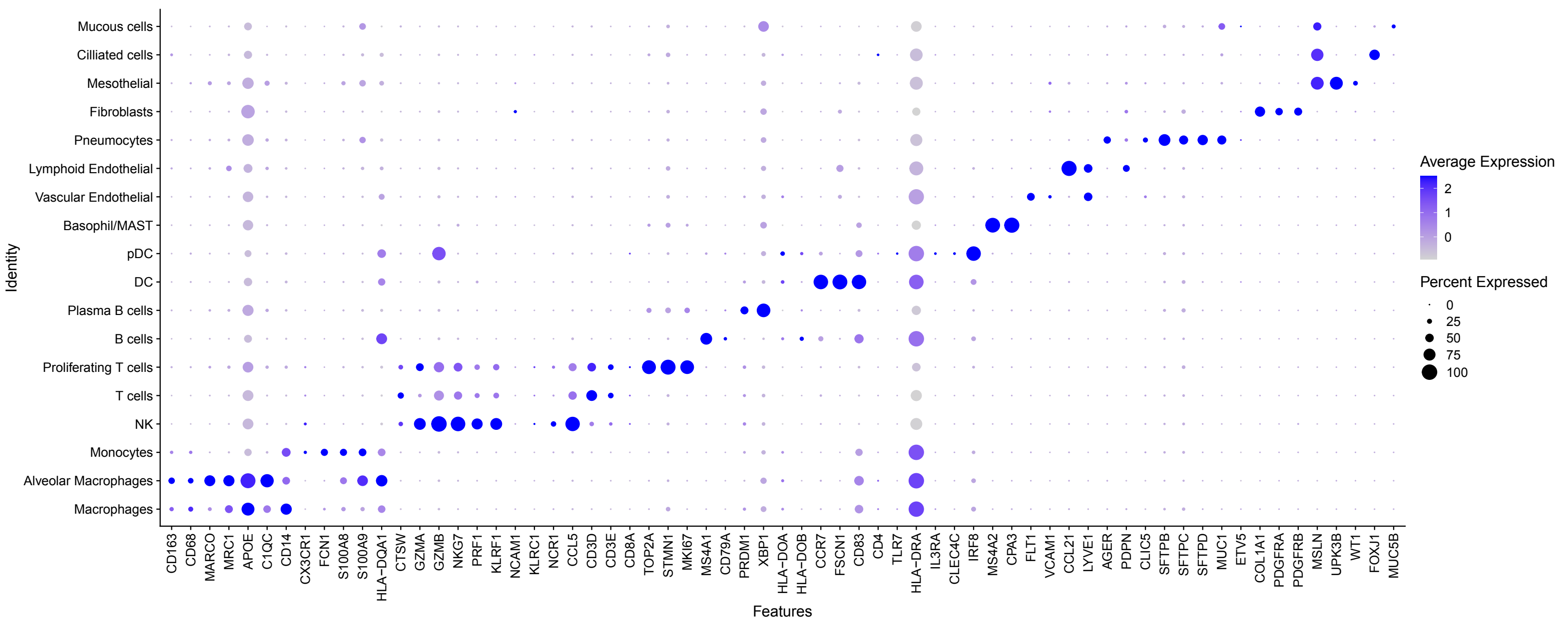

Supplementary Figure 5

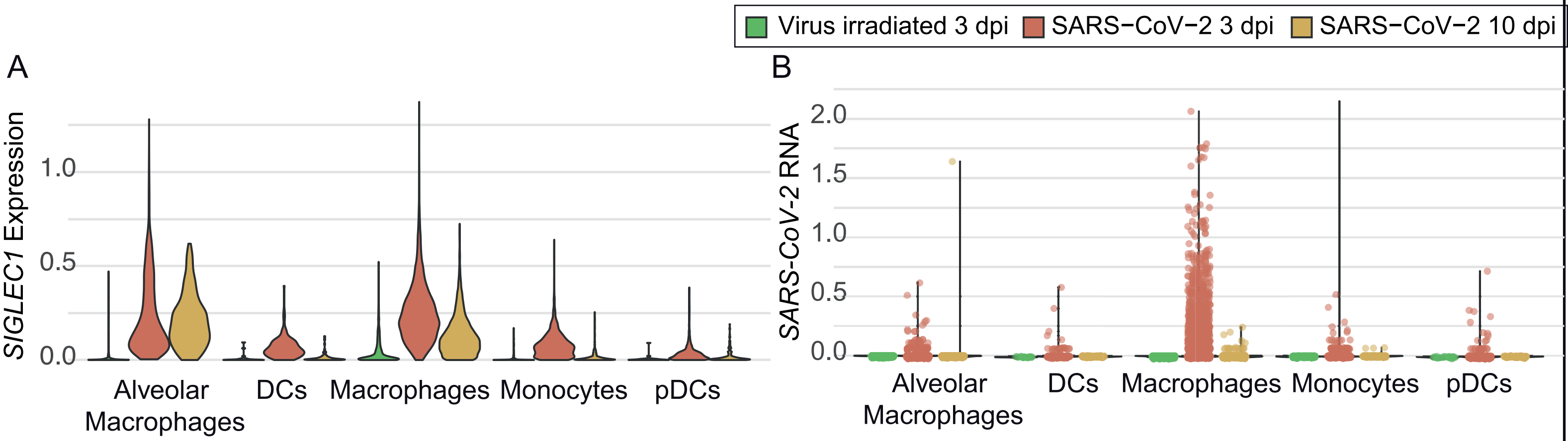

Supplementary Figure 6

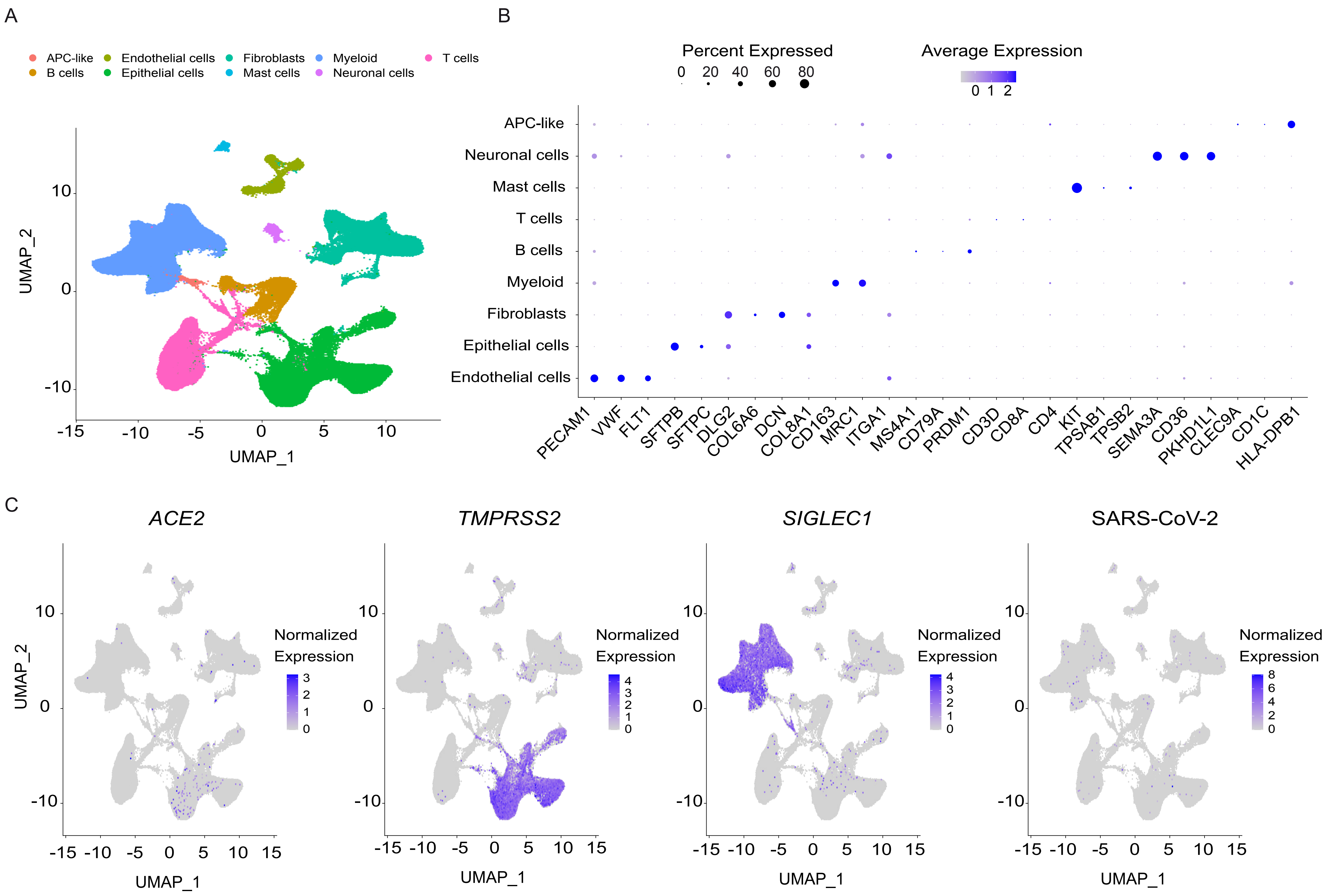

Supplementary Figure 7

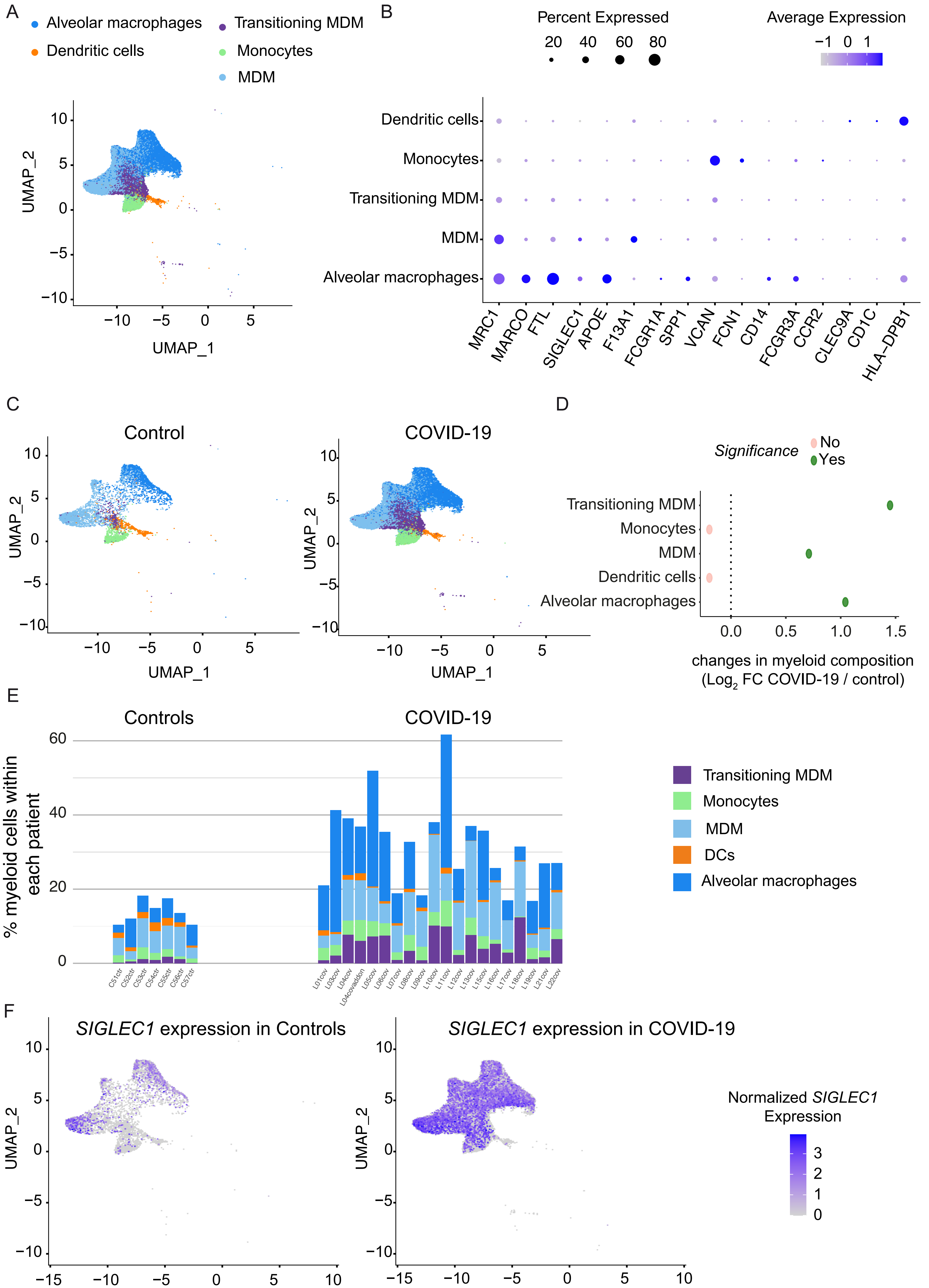

Supplementary Figure 8
